# Notes and News

**Published:** 1904-11-19

**Authors:** 


### NOTES AND NEWS.

A THEATRICAL reception will be held on November 22nd at St. James's Theatre, in aid of the Samaritan Free Hospital.

BY the will of the late Mr. Thomas Abbey Smithson, of Kingston-on-Hull, the Hull Royal Infirmary, the Dispensary, and Victoria Hospital for Children have been considerably benefited.

PRINCESS LOUISE AUGUSTA OF SCHLESWIG-HOLSTEIN, as lady president of the South Kensington district of the League of Mercy, held a meeting of lady vice-presidents last week, when it was decided to organise a dance in December on behalf of the League.

DR. GEORGE REID, commenting in his report on infantile mortality in Staffordshire, expresses a belief that it is hopeless to try and teach the mothers, and that the best course is to give school instruction, thus relying on the younger generation. For this proposal to be carried into effect the instruction first of teachers will be necessary.

THE death of Dr. Achille Vintras removes a distinguished and interesting man from the staff of the French Hospital in London. He was educated in England and Paris. He took his M.D. at St. Andrews in 1859. He was M.R.C.S. Eng. one year earlier. Later he was Resident Medical Officer at St. Mary's Hospital, and was finally Senior Physician to the French Hospital, which he was largely instrumental in founding. He acted as Physician to the French Embassy, was an Officer of the Legion of Honour, and a prolific writer, mainly on matters of climate and hygiene. He died at the age of 79. The funeral service was held at the Church of Notre Dame de France in Leicester Square, where a requiem mass was conducted. Numerous floral tributes were received, amongst them those from the French Ambassador, the Staff of the Embassy, the French Chamber of Commerce, and from many other French societies, and from his hospital. The French Ambassador attended the mass with members of the Embassy and many friends and relatives. Dr. Vintras was interred at the Brompton Cemetery.

A GRAND historical bazaar in aid of the Westminster Hospital is to be held in Dean's Yard, Westminster, three days in May. The stall-holders will wear historical costumes. The bazaar will be under the patronage of the Queen, and the stalls will be held by a large number of leaders of Society.

THE late Mr. Peter Mackinnon, of Argyllshire, has bequeathed £1,500 each to the Glasgow Royal Infirmary, the Glasgow Western Infirmary, the West of Scotland Convalescent Home, and the West of Scotland Association for the Relief of Incurables. He bequeathed £1,000 to the Victoria Infirmary, Glasgow, and £2,000 to the Cottage Hospital, Campbelltown.

ACCORDING to the *St. George's Hospital Gazette* a letter of thanks has been addressed to one of the surgeons there by the family of a patient who recovered from a "desperate attack of appendicitis." It began "Dear sir,—We are all so grateful that you have managed to do the next world out of our dear Susan." The same article contains a variety of gems extracted from the records of past cases. In one of them, "the abdomen was opened in the right iliac fossa, and on the outer side of the cæcum was found a little yellow puss, which was removed by irrigation and sponges on sticks."

THE Commission despatched to the Congo by the Liverpool School of Tropical Diseases to study sleeping sickness have sent in a report of their experiences, which cover an area of nearly 1,000 square miles. Cases of sleeping sickness appear to be present in nearly every town visited, from Leopoldville to Bompa. The members of the expedition have devoted special attention to finding a means of early detection of the disease, and this they seem to have been successful in doing. They report that in "cervical gland palpation" they have found a clinical method of detecting the commencement of the disease. The presence of the tsetse fly wherever the disease is prevalent is remarked upon.

THE new buildings of the Medical School and Laboratory at the Liverpool University were opened by the Earl of Derby and Lord Kelvin on Saturday. The new block is on the north side of the University quadrangle. The surgical theatre is constructed to accommodate 250 students, and rooms for the professors and other members of the teaching staff. The basement space is devoted to the study of practical surgery and ophthalmology; to anatomical museum, laboratory for materia medica and toxicology. Private laboratories for the professors are on the ground and first floors. The dissecting-room and anatomical theatre are on the first floor; on the next floor is the department for operative surgery, etc. The physics laboratory covers an area of 9,600 square feet. In the basement is a large workshop, a number of research rooms, and storage for various plant. The large lecture theatre is on the ground floor, as are also class-rooms, laboratories, apparatus-room. The first floor is devoted to senior students, and contains laboratories, workshop, library, and small rooms. The second floor contains research-rooms, photographic-rooms, and observatory. The total cost of the two buildings has been about £56,000.

